# The effect of proactive coping on posttraumatic growth among mobilized military personnel with various marital statuses after participating in combat operations

**DOI:** 10.3389/fpsyt.2026.1770239

**Published:** 2026-02-13

**Authors:** Ihor Prykhodko, Yanina Matsehora, Nataliia Kucherenko, Kateryna Marushchenko, Yurii Rumiantsev, Viktoriia Kuzina, Viktoriia Vintoniak

**Affiliations:** 1 Research Laboratory of Psychological Support of Service and Combat Activities of the National Guard of Ukraine, Educational and Scientific Institute for Personnel Work, National Academy of the National Guard of Ukraine, Kharkiv, Ukraine; 2 Department of Practical Psychology and Innovative Health Technologies, Educational and Scientific Institute “Ukrainian Engineering and Pedagogical Academy”, V. N. Karazin National University, Kharkiv, Ukraine; 3 Department of Military Psychiatry, Medical and Clinical Psychology, Ukrainian Military Medical Academy, Kyiv, Ukraine; 4 Department of Military Therapy, Ukrainian Military Medical Academy, Kyiv, Ukraine; 5 Department of General Military Disciplines, Ukrainian Military Medical Academy, Kyiv, Ukraine; 6 Department of Social Sciences, National Defense University of Ukraine, Kyiv, Ukraine

**Keywords:** combat operations, military personnel, posttraumatic growth, proactive coping, stress, mobilization, resilience, traumatization

## Abstract

**Introduction:**

The large-scale and intense combat actions that began in Ukraine on February 24, 2022, have necessitated an increasing mobilization of the civilian population for conscription into military service. Mobilized servicemen faced challenges in adapting to military service and the realities of intense combat. However, mobilization for military service also complicated the well-being of their families. The study aimed to determine the role of proactive coping in post-traumatic growth (PTG) among mobilized military personnel with various marital statuses after participating in combat operations.

**Methods:**

The Armed Forces of Ukraine mobilized military personnel (N = 237 males, aged 20–59 years) participated in this study after engaging in combat operations. The study participants were divided into two groups depending on their marital status: the married group and the unmarried group. The “Proactive Coping Questionnaire” and “Post-Traumatic Growth Inventory” were used to investigate the relationship between proactive coping and PTG among mobilized military personnel. Correlation and hierarchical linear regression analysis were used to determine the contribution of proactive coping to PTG and the role of marital status among mobilized military personnel.

**Results:**

The level of statistical significance in the married group was achieved between the coping strategy “Emotional Support Seeking” and the PTG domains “New Possibilities” (r = 0.310, p < 0.001), “Personal strength” (r = 0.325, p < 0.001), and “Post-traumatic Growth Overall Score” (r = 0.287, p < 0.001). The level of statistical significance was achieved in the unmarried group between the coping strategies “Reflective Coping” (r = 0.358, p < 0.001), “Preventive Coping” (r = 0.340, p < 0.001), “Instrumental Support Seeking” (r = 0.423, p < 0.001), and the PTG domain “New Possibilities”. The PTG domain “Relating to Others” showed a statistically significant correlation with the coping strategy “Emotional Support Seeking” (r = 0.347, p < 0.001). Such relationships were also found in the “Proactive Coping Overall Score” and the “Posttraumatic Growth Overall Score.”

**Discussion:**

Both married and unmarried service members showed similar average scores in terms of proactive coping and PTG after their combat experiences. Among married service members, PTG was linked solely to the coping style of “Seeking Emotional Support.” In contrast, unmarried service members exhibited PTG that was influenced by two proactive coping styles: “Reflexive Coping” and “Seeking Emotional Support.” Additionally, marital status played a role in moderating the impact of the overall proactive coping score on PTG, but it was a significant predictor only for unmarried service members. This research adds to the existing body of knowledge on personal growth induced by traumatic events and the role of proactive coping in PTG of mobilized military personnel with marital status. The results obtained lay the groundwork for future research that could enhance our understanding of this process among military personnel after combat operations.

## 1 Introduction

It is well-known that Russia invaded Ukraine on February 24, 2022, and this invasion has become the most serious military conflict in Europe since 1945. Hundreds of thousands of military personnel are engaged in combat operations on both sides of the war. Combat injuries received by Ukrainian service members were accompanied by particular severity, multiplicity, and combined defeat (1). Also, almost all military personnel participated in hostilities, experienced combat stress manifested in the form of acute stress reactions, affective and anxiety disorders, addictive and delinquent behavior, adjustment disorders, and suicidal manifestations (2–4). The demands on the mental health of military personnel have grown due to the circumstances, duration, and complexity of combat deployments (5). This has impacted their psychological resilience, behavior during these experiences, their coping mechanisms for managing combat stress and recovering from trauma (6, 7).

The duration and intensity of the war exacerbated the problem of mobilizing civilians and training military personnel for combat. After more than three years of full-scale war, the vast majority of Ukrainian combatants were mobilized servicemen. They had not chosen military service as a career path or aspired to dedicate their lives to military duties. Instead, they were compelled to defend their families, their way of life, democratic values, and their country. These servicemen faced challenges in adapting to military service and the realities of intense combat (2). They had to navigate the challenges of war with only brief military training that lasted up to two months, while also drawing on their previous civilian experience and personal traits. As a result of these challenges and other factors, many servicemen left the combat zone without permission (8).

However, mobilization for military service also complicated the well-being of their families (9). Family support has been shown in previous studies to be a valuable resource for combat-deployed military personnel in coping with stressors, and having a strong emotional bond with a spouse acts as a protective buffer (10, 11). If this support channel is active, other strategies, such as seeking support from colleagues or professional help, may be redundant (12). For married military personnel, marital quality is often a stronger predictor of the absence of PTSD and depression than their own individual coping skills (13). However, Ukrainian families faced problems related to threats to family members’ health and lives, shifts in family routines, and daily challenges on their own, without men/women (9). This was also due to combat operations in the country, which involved constant threats of missile attacks, loss of housing, forced displacement, distance learning for children, and their prolonged stay at home, as well as remote work or lack thereof, etc. (14). Families of mobilized service members often struggled to provide effective emotional support to their loved ones, and in some cases, even exacerbated their mental health issues by misreporting the challenges they faced in their absence (9). These situations contributed to the breakdown of family relationships. They added to the stress experienced by mobilized service members, who, while defending their country, were unable to protect and support their families.

Coping with potentially traumatic events, such as combat stress, involves utilizing various coping mechanisms and strategies (15). Traditional stress research typically examines how individuals respond to stress when they experience it directly (16, 17). Other studies focus on actions that can be taken before a stressful event occurs (18). This approach to coping emphasizes personal growth and self-regulated strategies, encouraging a proactive, goal-oriented, and adaptive way of managing stress (19–21). This involves developing resources and acquiring skills that may not be immediately necessary for addressing the current threat, but which will help prepare for potential future threats (22). Proactively addressing the threat through emotions, thoughts, and behavior helps combat them in their early stages instead of dealing with the aftermath of a full-blown trauma (23, 24).

Historically, research of stress has focused on negative outcomes following trauma and adversity; in particular, on the development of maladaptive conditions post-trauma, including both mental and physical disorders (25). Nevertheless, a growing number of studies indicate that having to cope with trauma can result in post-traumatic growth (PTG) (26–28). Some studies view growth as the result of a person’s ongoing efforts to reinterpret a traumatic event and cope with related distress (29, 30). Others associate personal growth with the search for meaning after trauma (31), while some describe it as a compensatory illusion (32, 33). Thus, the term PTG is commonly used to describe the positive changes that can occur after a person faces a significant life crisis (28). Such changes can manifest in five main directions: an increased appreciation for life, a deeper meaning in relationships, a greater sense of personal empowerment, a shift in priorities, and a more enriched spiritual life (26, 34, 35).

PTG characteristics have been studied among (ex-)military personnel to determine evidence of growth and its dynamics, as well as whether this growth is associated with sociodemographic, military, trauma, or mental health factors (36). Notably, military personnel who experience PTG readapt to military life and demonstrate functional improvement, thereby contributing to the combat power of their unit (37). It was found that a more thorough understanding of PTG among military personnel may also have implications for clinical practice, by confirming whether PTG should be incorporated into psychological treatments for service members and veterans (33). Indeed, programs and training, such as “Comprehensive Soldier Fitness”, “Higher Ground”, and “Battlemind”, which help facilitate well-being, resilience, and decompression in post-deployment military personnel, are starting to acknowledge PTG (37).

Thus, the identified research gap lies in the absence of empirical studies exploring the synergistic relationship between proactive coping strategies and PTG among mobilized service members exposed to stress factors of a long-term full-scale war. Investigating the interaction between proactive coping, PTG, and marital status in this population is a novel and necessary contribution for the following reasons. 1) Unlike professional soldiers, these individuals are “forced” combatants who draw on civilian life experiences rather than long-term military socialization. 2) While traditional research often views the family as a support system, during a long war, the family is also an additional stressor due to missile attacks, displacement, and frequent relationship breakdown. 3) There is a lack of data on how proactive coping influences the formation of PTG in mobilized military personnel after participating in combat operations. 4) Understanding this interaction is necessary to refine psychological training and recovery programs for military personnel.

We hypothesized that 1) proactive coping would be positively associated with PTG, and 2) marital status would moderate this relationship, with the association being stronger for married personnel.

The study aimed to determine the role of proactive coping in post-traumatic growth among mobilized military personnel with various marital statuses after participating in combat operations.

## 2 Materials and methods

### 2.1 Study design and participants

The study was an exploratory descriptive design. The military personnel of the Armed Forces of Ukraine (N = 237 males, aged 20–59 years) participated in this study. All military personnel were mobilized for military service between 2022 and 2025. After participating in combat operations, due to worsening mental and physical conditions, reduced adaptive abilities, and increased posttraumatic stress (PTS) symptoms, they were sent to a two-week psychological recovery program at the rehabilitation center (38). The rehabilitation center was located 30–40 km from the combat zone. This program began in June 2022 and continues to this day, based on the clinical sanatorium in the Kharkiv region. The psychological recovery program for military personnel was specially developed for its practical implementation: we named it the “Invincibility Program.” The purpose of this program was to reduce the impact of PTS on combatants, strengthen mental health and mobilize their psychological resources, improve adaptation and resilience, and promptly return to combat activities. The main activities included in the “Invincibility Program” were carried out in a group form and consisted of three sections: psychological, medical, and social events (38). After completing this program, all servicemen returned to the combat zone to continue performing tasks.

Participants for the study were chosen using consecutive sampling from the rehabilitation center. The participants were identified without a specific symptom profile with various manifestations of acute stress reactions; significant negative experiences, including signs of depression and suicidal ideation; sleep problems; somatic complaints; wounds and contusions; difficulties in returning to combat missions due to the consequences of illness, injury, and wounds. Female military personnel were not included in this study because, over the entire period of the program, less than 0.5% of female combatants participated. All study participants were divided into two groups depending on their marital status: group 1 (married) and group 2 (unmarried). **Table 1** presents the socio-demographic composition of the sample.

**Table 1:** The socio-demographic composition of the sample, (n, %).

Attribute		
Military rank	Private soldiers	179 (75.5)
	NCO	58 (24.5)
Combat experience (months)	less than 3	28 (11.8)
	4-5	71 (30)
	more than 6	138 (58.2)
Age	20-29	31 (13.1)
	30-39	60 (25.3)
	40-49	101 (42.6)
	50-59	45 (19)
Marital status	Married/Common-law marriage	151 (63.7)
	Unmarried/Divorced/Widowers	86 (36.3)
Education	Higher	27 (11.4)
	Secondary technical	23 (9.7)
	Vocational	65 (27.4)
	Secondary	108 (45.6)
	Incomplete secondary	14 (5.9)

### 2.2 Measures

The study utilized psychodiagnostic tools to explore the connection between proactive coping and PTG among mobilized military personnel with various marital statuses after participating in combat operations.

The “Proactive Coping Questionnaire” (PCQ) was utilized to examine the characteristics of proactive coping strategies employed by mobilized military personnel in addressing combat stress (39). The PCQ is a modified short version of the “Proactive Coping Inventory” (PCI) (24). The PCQ was modified and adapted using a sample of Ukrainian military personnel undergoing psychological recovery after participating in combat operations in rehabilitation centers. This modification reduced the number of items in the PCQ. The PCQ contains 35 items covering cognitive, behavioral, and emotional coping strategies based on resourcefulness, responsibility, and foresight. The PCQ uses a four-point Likert scale (zero = “strongly disagree,” three = “strongly agree”). The average of seven subscales scores yielded an overall level of proactive coping, with higher scores indicating greater proactive coping. The Cronbach’s α coefficients established for the PCQ are as follows: “Proactive Coping” 0.741; “Reflection Coping” 0.751; “Strategic Planning” 0.743; “Preventive Coping” 0.739; “Instrumental Support Seeking” 0.610; “Emotional Support Seeking” 0.778; “Avoidance Coping” 0.776; “Overall Proactive Coping Score” 0.889. Considering the strong Cronbach’s α values for the “Overall Proactive Coping Score” scale (0.889) and the intention to maintain the original author’s structure of seven scales (24), it was decided to retain the “Instrumental Support Seeking” scale, even though it had minimally acceptable values (0.610).

The “Post-traumatic Growth Inventory” (PTGI) (34) was utilized to examine the level of PTG among mobilized military personnel following their participation in combat operations. The PTGI is measured by 21 items across five domains: “Relating to Others”, “New Possibilities”, “Personal Strength”, “Spiritual Change”, and “Appreciation of Life”. The PTGI uses a six-point Likert scale (zero = “After the events, I didn’t experience any changes,” five = “After the events, I experienced huge changes”). The average of five domain scores yielded an overall level of PTG, with higher scores indicating greater PTG. The Ukrainian adaptation of the PTGI (40) demonstrated high reliability: Cronbach’s α 0.91.

### 2.3 Statistical analysis

Correlation analysis was used to determine the relationship between proactive coping measures and PTG in married and unmarried mobilized military personnel. The Bonferroni correction (significance level α = 0.05 (48 comparisons)) was used to control for type I errors in multiple correlation analysis. Hierarchical linear regression analysis was used to determine the role of military marital status and the contribution of proactive coping to PTG. The Simple Slopes Plot allowed us to determine the relationship between the overall proactive coping score and PTG scores separately for each marital status category (married or unmarried). We included the variables “marital status (0 – unmarried, 1 – married)” and “interaction term marital status*proactive coping” in the regression analysis as independent variables in addition to proactive coping to assess the moderating effect of marital status. To represent the data, we used the main descriptive statistics (M, SD). The reliability of differences in the results of the mean values in two interrelated groups was determined using the Student’s t-test. To assess the statistical significance of differences, we used the significance level p < 0.05. The statistical analysis of the study results was carried out using the program SPSS 22.0 (IBM, Armonk, NY, USA).

## 3 Results

The results indicated that participants with varying marital statuses exhibited similar average scores for proactive coping and all domains of PTG (**Table 2**).

**Table 2:** Proactive coping and PTG scores for participants with different marital statuses.

Variables	General sample (N = 237)	Groups		Differences between groups	
		Group 1 (n_1_ = 151)	Group 2 (n_2_ = 86)	t	p
“Proactive Сoping Questionnaire”					
Proactive Coping	8.86 ± 3.14	8.89 ± 3.06	8.79 ± 3.29	0.243	0.808
Reflective Coping	9.32 ± 3.19	9.28 ± 3.23	9.37 ± 3.13	0.202	0.840
Strategic Planning	8.88 ± 3.56	8.92 ± 3.74	8.80 ± 3.24	0.245	0.807
Preventive Coping	9.64 ± 3.18	9.76 ± 3.18	9.42 ± 3.18	0.798	0.425
Instrumental Support Seeking	8.74 ± 3.17	8.59 ± 3.09	9.01 ± 3.29	0.986	0.325
Emotional Support Seeking	9.34 ± 3.55	9.25 ± 3.31	9.51 ± 3.95	0.555	0.580
Avoidance Coping	8.24 ± 3,03	8.17 ± 3.11	8.37 ± 2.89	0.488	0.626
Proactive Coping Overall Score	46.53 ± 14.51	46.52 ± 14,58	45.53 ± 14.48	0.006	0.995
“Post-traumatic growth inventory”					
Relating to Others	17.22 ± 8.40	16.61 ± 8.33	18.12 ± 8.46	1.475	0.142
New Possibilities	12.61 ± 6.51	12.12 ± 6.59	13.47 ± 6.31	1.534	0.126
Personal Strength	10.43 ± 5.30	10.25 ± 5.18	10.73 ± 5.53	0.670	0.503
Spiritual Change	4.4 ± 3.72	4.87 ± 4.05	5.06 ± 3.08	0.365	0.715
Appreciation of Life	8.39 ± 4.14	8.35 ± 4.03	8.44 ± 4.36	0.150	0.881
Post-traumatic Growth Overall Score	53.58 ± 24.59	52.21 ± 24.79	55.98 ± 24.17	1.134	0.258

All proactive coping measures had similar average levels in both groups. Some increases were seen in the following scales: “Preventive Coping”, “Emotional Support Seeking”, and “Reflective Coping”. However, they had different correlations between proactive coping and PTG (**Tables 3**, **4**).

**Table 3:** Correlation indicators between the “Proactive Coping Questionnaire” and the “Post-traumatic Growth Inventory” in married participants (n_1_ = 151).

Variables		Proactive coping	Reflective coping	Strategic planning	Preventive coping	Instrumental support seeking	Emotional support seeking	Avoidance coping	Proactive coping overall score
Relating to Others	r	-0.012	0.022	-0.070	0.020	0.176	0.231	0.150	0.047
	p	0.885	0.791	0.393	0.806	0.031	0.004	0.066	0.570
New Possibilities	r	0.069	0.104	0.002	0.098	0.241	0.310^*^	0.187	0.141
	p	0.397	0.202	0.981	0.233	0.003	0.001	0.022	0.083
Personal Strength	r	0.057	0.057	0.004	0.094	0.201	0.325^*^	0.183	0.124
	p	0.488	0.484	0.960	0.253	0.013	0.001	0.024	0.130
Spiritual Change	r	0.158	0.186	0.140	0.140	0.249	0.165	0.232	0.182
	p	0.053	0.022	0.087	0.085	0.002	0.043	0.004	0.025
Appreciation of Life	r	0.045	0.041	0.016	0.080	0.090	0.197	0.100	0.083
	p	0.580	0.619	0.841	0.328	0.273	0.015	0.222	0.313
Post-traumatic Growth Overall Score	r	0.060	0.084	0.003	0.088	0.221	0.287^*^	0.192	0.122
	p	0.468	0.304	0.967	0.281	0.006	0.001	0.018	0.135

*p < 0.001 (Bonferronni correction for 48 comparisons).

**Table 4:** Correlation indicators between the “Proactive Coping Questionnaire” and the “Post-traumatic Growth Inventory” in unmarried participants (n_2_ = 86).

Variables		Proactive coping	Reflective coping	Strategic planning	Preventive coping	Instrumental support seeking	Emotional support seeking	Avoidance coping	Proactive coping overall score
Relating to Others	r	0.257	0.332	0.229	0.212	0.312	0.347^*^	0.078	0.378^*^
	p	0.017	0.002	0.034	0.050	0.003	0.001	0.474	0.001
New Possibilities	r	0.308	0.358^*^	0.239	0.340^*^	0.423^*^	0.303	0.188	0.417^*^
	p	0.004	0.001	0.027	0.001	0.001	0.005	0.083	0.001
Personal Strength	r	0.188	0.253	0.181	0.227	0.309	0.294	0.093	0.320
	p	0.083	0.019	0.096	0.036	0.004	0.006	0.395	0.003
Spiritual Change	r	0.087	0.093	0.117	0.062	0.204	0.304	0.099	0.189
	p	0.425	0.394	0.284	0.568	0.059	0.004	0.363	0.081
Appreciation of Life	r	0.258	0.269	0.215	0.323	0.218	0.255	0.094	0.336
	p	0.016	0.012	0.047	0.002	0.044	0.018	0.389	0.002
Post-traumatic Growth Overall Score	r	0.271	0.328	0.238	0.281	0.356^*^	0.353^*^	0.127	0.399^*^
	p	0.012	0.002	0.028	0.009	0.001	0.001	0.242	0.001

*p < 0.001 (Bonferroni correction for 48 comparisons).

Bonferroni correction in group 1 showed that the level of statistical significance was achieved in three correlations between the coping indicators “Emotional Support Seeking” and the PTG domains “New Possibilities”, “Personal strength”, and “Post-traumatic Growth Overall Score”. Bonferroni corrections in group 2 showed that the level of statistical significance was achieved between the coping strategies “Reflective Coping”, “Preventive Coping”, “Instrumental Support Seeking”, and the PTG domain “New Possibilities”. Furthermore, the PTG domain “Relating to Others” showed a statistically significant correlation with the coping strategy “Emotional Support Seeking”. Such relationships were also found in the “Proactive Coping Overall Score” and the “Post-traumatic Growth Overall Score”. Statistical analysis confirmed our first hypothesis of a significant positive correlation between the use of proactive coping strategies and the level of PTG. Thus, participants who are prone to anticipating stressful events and accumulating personal resources to overcome them demonstrated higher rates of positive personal change after experiencing combat stress.

A hierarchical linear regression analysis was conducted to determine the role of marital status in the contribution of proactive coping to PTG. Eight separate analyses were performed, based on the number of proactive coping behaviors and the proactive coping overall score. In the first step, all proactive coping variables were included in each model (0 – unmarried, 1 – married). Statistical significance (F, p, R^2^, R^2adjusted^) was adjusted for the models built for the different proactive coping measures and the proactive coping overall score (**Appendix А**). At this stage, the beta coefficients for the interaction term were significant, showing that marital status moderates the relationship between proactive coping (both specific coping strategies and the general indicator of proactive coping) and PTG. **Appendix B** presents the beta coefficients in the models of relationships between PTG and various proactive coping. For the “Proactive Coping Overall Score” model, the “interaction term” variable had a statistically significant beta coefficient (b = -0,471, β = -0,486, t = -2,116, p = 0,035). Thus, marital status moderates the relationship between the “Proactive Coping Overall Score” and PTG.

**Figure 1:**
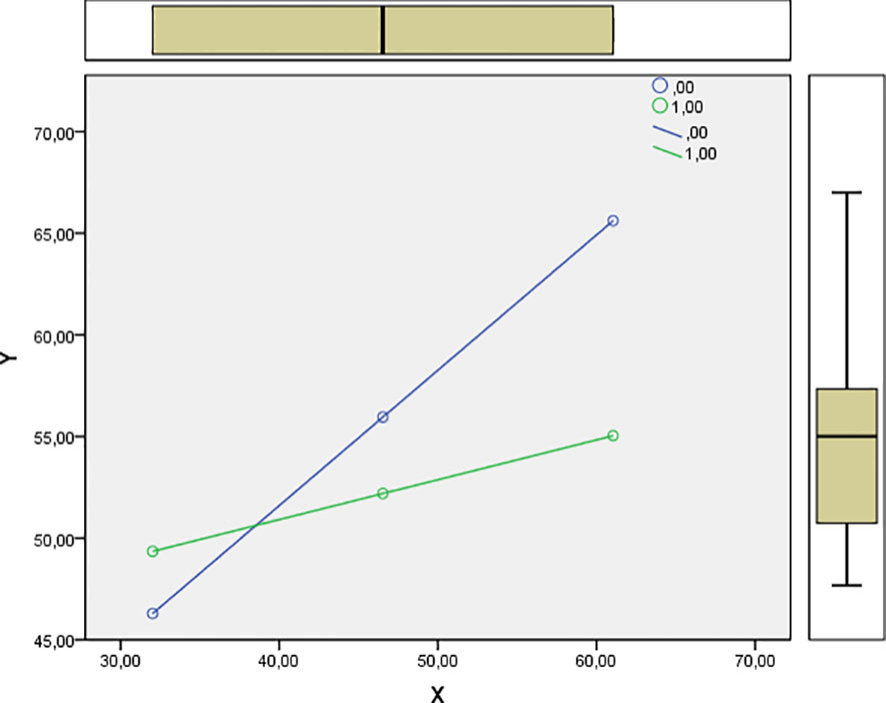
Moderation plot: visualization of the relationship between the “Proactive Coping Overall Score” and PTG for married and unmarried participants. X is the “Proactive Coping Overall Score”, Z is marital status (0 = unmarried participants, 1 = married participants), and Y is PTG.

The models were added “interaction term marital status*proactive coping” at the second stage. In this step of studying the effect of the relationship between the “Proactive Coping Overall Score” and marital status on the development of PTG, the Simple Slopes Plot was conducted. The results indicated a statistically significant relationship between proactive coping and PTG among unmarried military personnel (β = 0.396, t = 3.756, p = 0.001). This suggests that higher levels of proactive coping are associated with greater PTG for this group. In contrast, for married military personnel, the relationship between proactive coping and PTG was found to be statistically insignificant (β = 0.116, t = 1.457, p = 0.146). **Figure 1** provides a graphical representation of this relationship.

Thus, marital status moderated the contribution of proactive coping to PTG in study participants. But the second hypothesis, that marital status moderates the relationship between proactive coping and PTG, while strengthening it in married military personnel, was not supported. In fact, the opposite effect was found: a statistically significant moderation was found only in the group of single (unmarried) military personnel.

Although the “interaction term” variable for various coping styles was not statistically significant, this does not mean that these coping styles do not play a role in predicting PTG in each group individually. Below are the regression equations demonstrating that coping styles are significant predictors of PTG for each group.


$${\text{PTG}}_{\text{unmarried}}=20,027+2,017\text{RC}+1,792\text{ESS}$$

where PTG – Post-traumatic Growth; 20,027 – Сonstant; RC – Reflective Coping; ESS – Emotional Support Seeking.

For this model: F = 9.66, p = 0.001, R^2^ = 0.189 та R^2^_adjusted_ = 0.169.


$${\text{PTG}}_{\text{married}}=32.367+2.159\text{ESS}$$

where PTG – Post-traumatic Growth; 32.367 – Сonstant; ESS – Emotional Support Seeking.

For this model: F = 13.314, p = 0.001, R^2^ = 0.084 та R^2^_adjusted_ = 0.078.

The regression equations indicate that for unmarried participants, higher levels of “Emotional Support Seeking” and “Reflective Coping” behaviors are associated with increased PTG. Specifically, the regression model for the unmarried group explains approximately 17% of the variance in PTG. In contrast, among married participants, only “Emotional Support Seeking” coping demonstrates a statistically significant impact on PTG. The regression model for married participants accounts for less than 8% of the variance in PTG.

## 4 Discussion

The study explored the connection between proactive coping and PTG in mobilized military personnel subjected to the stressors of a prolonged, full-scale war. The findings showed that in both groups, all proactive coping strategies were moderately pronounced. The average rates of PTG in our study indicate that, although individuals experience similar traumas, there is no single route to personal growth. Each type of trauma may lead to various forms of growth and different levels of resource availability (41, 42). It’s important to note that our sample was diverse in terms of resources for PTG. We believe that proactivity influences PTG in multiple ways, depending on factors beyond just marital status. However, due to the small sample size, we were unable to test this hypothesis.

It is important to note that our study sample consisted of mobilized military personnel participating in a recovery rehabilitation program. They exhibited signs of maladaptation, PTS symptoms, physical fatigue, sleep issues, and other related challenges. These peculiarities may have hindered their ability to experience PTG during this period. Previous research has also highlighted the significance of considering the time elapsed since the traumatic event when assessing PTG (43). Our results indicated that not all proactive coping had an impact on PTG, while emotional support seeking appeared to play a significant role in fostering such growth. These results were affected by the particular characteristics of the study sample. Furthermore, the absence of a significant moderator effect of marital status may be a consequence of the insufficient sample representativeness, which did not allow for the full verification of differences in the mechanisms of proactive coping among participants with different marital statuses. Also, the lack of statistical significance is primarily attributed to the high stringency of the Bonferroni correction and specific external variables, which were outside the scope of this study but represent a crucial area for future research.

The observed moderation of proactive coping and PTG, which was more pronounced in unmarried military personnel, can be explained by the following psychological mechanisms. Married military personnel benefit from a powerful and valuable external resource: family social support (44, 45). In stressful situations, they can rely on their spouses, making their PTG less dependent on internal coping skills. Unmarried military personnel may have limited external support resources, so their PTG directly and critically depends on their ability to engage in proactive behavior. For them, proactivity can be the main driver of growth. Unmarried military personnel also have greater autonomy in planning for the future and changing life goals after trauma. Proactive, future-oriented coping is more easily achieved when a person does not need to reconcile their life transformations with family interests. This may lead to a greater sense of personal growth through the realization of one’s own plans. The absence of a family may force unmarried service members to develop proactive cognitive skills more intensively. In this case, proactive coping becomes a key adaptation tool, and each success in this area leads to a more significant increase in PTG than in married service members. It can be hypothesized that married service members may more often experience PTG in their relationships, which naturally occurs through family. Meanwhile, for unmarried service members, growth is more associated with discovering “New Possibilities” and “Personal Strength”: precisely the components most closely associated with proactive coping.

In our study, we found that married participants showed almost no difference in PTG compared to unmarried military personnel. PTG in both groups was found to be influenced by the coping style “Emotional Support Seeking.” “Reflective Coping,” which involves internally processing and reflecting on the situation, contributed to increased PTG among unmarried participants. Similar results were found in a study measuring PTG in military personnel, which indicated that higher levels of social support, spirituality, and rumination were linked to increased PTG (36). Social support, spirituality, and a tendency toward reflection contribute to PTG in military personnel, which is based on the concept of positive psychological changes following critical events. These factors complement each other: reflection initiates the process of processing experience; spirituality provides direction and a meaningful framework for this processing; and social support provides emotional resources and confirms the correctness of the new path. It has previously been shown that social support is the most stable predictor of PTG (37). In the context of military service, this includes support from colleagues, family, and society (11). Greater social support was significantly associated with better dyadic functioning (44). Having a safe environment allows service members to discuss traumatic experiences openly (46). Collectivism and military brotherhood reduce feelings of isolation (36). As a result, social approval of new life meanings reinforces positive changes, transforming individual trauma into a collective experience of overcoming.

Despite the model’s limitations, it was established that among unmarried military personnel, the proactive coping “Emotional Support Seeking” and “Reflective Coping” explain up to 17% of the variance in PTG scores. These copings are significantly linked to “Relating to Others” and the pursuit of “New Possibilities.” For social-humanitarian research, such a result represents an acceptable indicator (47). This provides grounds for developing targeted psychological recovery programs for unmarried military personnel. Such programs should utilize cognitive behavioral therapy techniques to stimulate conscious reflection on traumatic experiences. The focus of therapy should shift from rumination (intrusive thoughts) to an active search for new meanings and opportunities in life. In working with unmarried servicemen, it is essential to address and modify cognitive distortions regarding emotional vulnerability. Psychotherapeutic efforts should focus on internalizing the concept that trust and seeking support are manifestations of psychological maturity and resource expansion, rather than signs of weakness or “failure” of masculinity. Consequently, fostering proactivity, planning skills, preventative coping with stress, and seeking social support can be an effective target in psychological counseling of mobilized military personnel. This approach aims to prevent PTSD and promote PTG, irrespective of their marital status.

It can be hypothesized that the identified moderator effect of marital status on the contribution of proactive coping to PTG is associated not only with the social support provided by spouses, but rather with the collectivist or individualistic stress-coping culture. Previous studies also recommend considering proactive coping through the lens of collectivist and individualistic cultures (48). However, we will only be able to test this hypothesis in future studies.

Future research would be useful to focus on clarifying the role of various proactive coping in fostering PTG, especially in groups with enhanced opportunities for proactive growth, such as individuals after military demobilization. Additionally, it would be valuable to explore how proactivity contributes to specific aspects of PTG in military personnel and veterans, including awareness of new opportunities, personal strength, and attitudes toward others.

This study certainly had limitations. 1) The study served as a generalization of experiences from the rehabilitation center focused on the psychological recovery of military personnel following participation in hostilities. This significantly influenced the amount of available empirical data, including sample size and psychodiagnostic survey methods, the study’s structure, and the mathematical processing methods used. 2) Female military personnel were excluded from this study because less than 0.5% of female combatants participated throughout the duration of the psychological recovery program. 3) The participant sample consisted solely of enlisted military personnel and sergeants; officers did not take part in this study. 4) The research was an exploratory and descriptive design, aimed to identify recovery resources for military personnel who, after mobilization and short-term basic military training, were deployed to intense combat zones. It is also important to note that all study participants returned to their units for further combat. Thus, the time factor played a significant role in shaping the identified characteristics of PTG and the influence of proactive coping on its development. 5) Considering the strong Cronbach’s α values for the overall PCQ scale and the intention to maintain the original author’s structure of seven scales, it was decided to retain the “Instrumental Support Seeking” scale, even though it had minimally acceptable values (0.610). 6) Existing research on family support for servicemen relates to scenarios where families are safe, a situation not observed in Ukraine, creating an ambivalent role for the family that can serve as both a source of anxiety and a resource. 7) The research was conducted on a sample of servicemen who participated in the psychological recovery program, specifically those exhibiting signs of mental and physical exhaustion, PTS symptoms, and maladjustment. Consequently, the results related to the characteristics of proactive coping and PTG in both established variants—typical for married and single mobilized servicemen—should be specifically attributed to the corresponding samples. Therefore, it is inappropriate to extend the findings of this study to servicemen who cope more effectively with the impact of combat stress. 8) The calculated coefficient of determination (R^2^) for the marital status moderator model is relatively low, indicating the presence of a significant number of unaccounted-for factors. This is attributed to the high heterogeneity of the sample of mobilized military personnel, who have different life experiences of overcoming traumatic situations, distinct cognitive strategies, and resilience resources. Another important factor is the subjective perception of family roles, which extends beyond formal status (i.e., being married or unmarried). Therefore, generalizing the role of proactivity in PTG requires further typological research using cluster analysis to identify specific groups of respondents. 9) The acute phase of recovery post-combat, in rehabilitation, may not be the optimal time to capture stable PTG. Another important factor influencing the development of PTG is the return of military personnel to the combat zone to continue performing combat missions. 10) This study faced limitations due to the brief duration of the psychological recovery program and the need to avoid overloading participants with additional activities that did not align with the program’s objectives, which limited the application of research methods and the repeatability of the survey, among other factors. Finally, the current study was constrained by the absence of an active comparison condition and a longitudinal follow-up. Thus, by summarizing some of the outcomes from the rehabilitation center’s work on the psychological recovery of military personnel after combat operations, we aimed to attract the scientific community’s attention to addressing new urgent challenges in military medicine and psychology, particularly in the unique area of psychological support for mobilized military personnel.

## 5 Conclusion

The mental health of mobilized military personnel occupies an important place in psychological support for combat operations. This research adds to the existing body of knowledge on personal growth induced by traumatic events and the role of proactive coping in post-traumatic growth of mobilized military personnel with marital status.

Both married and unmarried service members showed similar average scores in terms of proactive coping and posttraumatic growth after their combat experiences. Among married service members, posttraumatic growth was linked solely to the coping style of “Seeking Emotional Support.” In contrast, unmarried service members exhibited posttraumatic growth that was influenced by two proactive coping styles: “Reflexive Coping” and “Seeking Emotional Support.” Additionally, marital status played a role in moderating the impact of the overall proactive coping score on posttraumatic growth, but it was a significant predictor only for unmarried service members.

The results obtained lay the groundwork for future research that could enhance our understanding of this process among military personnel after combat operations. Although the statistical data obtained indicate a limited practical impact, they offer opportunities for more focused research. Specifically, a promising area for enhancing military recovery programs is to better understand the role of emotional support in fostering and promoting post-traumatic growth.

## Data Availability

The dataset for this article is not publicly available due to ethical and privacy concerns. Requests to access the datasets should be directed to the corresponding author.

## References

[1] Shvets AV Marushchenko KY Poliukhovych VI Pudaіlo MP . Features of the information factor influence on mental health characteristics of servicemen after participation in combat actions. Ukr J Mil Med. (2024) 5:24–31. doi: 10.46847/ujmm.2024.1(5)-024

[2] Kalnysh VV Serheta IV Pashkovskiy SM Shvets AV . Features of the formation of behavioral reactions of servicemen at the combat zone under the influence of socio-psychological factors. Ukr J Mil Med. (2025) 6:58–68. doi: 10.46847/ujmm.2025.3(6)-058

[3] Kokun O Pischko I Lozinska N . Examination of military personnel’s changed psychological states during long-term deployment in a war zone. Psicol. (2022) 38:191–200. doi: 10.6018/analesps.475041

[4] Prykhodko I Matsehora Y Kryvokon N Hunbin K Kovalchuk O Antusheva N . Manifestations of post-traumatic stress in military personnel after participating in hostilities in the Russian-Ukrainian war. Eur J Clin Exp Med. (2023) 21:776–84. doi: 10.15584/ejcem.2023.4.19

[5] Hukovskyy O West JC Morganstein JC Augusterfer EF Benedek DM Boyko O . The combat path: sustaining mental readiness in ukrainian soldiers. US Army War Coll Q Parameters. (2024) 54. doi: 10.55540/0031-1723.3285

[6] Kokun O Pischko I Lozinska N Stasiuk V Oliinyk V . Mental health of servicemen, POWs, and civilians in Ukraine: A comparative study. J Loss Trauma. (2024), 1–26. doi: 10.1080/15325024.2024.2411694

[7] Prykhodko I Kolesnichenko O Matsehora Y Aleshchenko V Kovalchuk O Matsevko T . Effects of posttraumatic stress and combat losses on the combatants’ resilience. Cesk Psychol. (2022) 66:157–69. doi: 10.51561/cspsych.66.2.157

[8] Pashchenko EM . Criminality of military servants of the army and other military forms of Ukraine in the conditions of Russian aggression against Ukraine. Criminology aspect. Uzhhorod Natl Univ Herald Ser Law. (2024) 2:324–9. doi: 10.24144/2307-3322.2024.81.2.50

[9] Matsehora Y Kucherenko N Kryvokon N Filonenko V Herasymenko O Kramchenkova V . The impact of combat stress on the coping strategies selected by mobilized military personnel with differing family statuses. Rom J Mil Med. (2025) 128:446–54. doi: 10.55453/rjmm.2025.128.5.7

[10] Green S Nurius PS Lester P . Spouse psychological well-being: A keystone to military family health. J Hum Behav Soc Environ. (2013) 23:753–68. doi: 10.1080/10911359.2013.795068, PMID: 24415897 PMC3885258

[11] Na PJ Tsai J Southwick SM Pietrzak RH . Provision of social support and mental health in U.S. military veterans. NPJ Ment Heal Res. (2022) 1:4. doi: 10.1038/s44184-022-00004-9, PMID: 38609471 PMC10938859

[12] Sprague-Jones J McKenney K Firman C Susskind Y Bash K . Development of the protective factors Survey, military families (PFS-MF). Child Youth Serv Rev. (2025) 169:108131. doi: 10.1016/j.childyouth.2025.108131

[13] Wesemann U Rowlands K Renner KH Konhäuser L Köhler K Himmerich H . Impact of life-threatening military incidents during deployments abroad on the relationships between military personnel and their families. Front Psychiatry. (2024) 15:1419022. doi: 10.3389/fpsyt.2024.1419022, PMID: 39091456 PMC11291243

[14] Kostenko A Semenov V Osetrova O Kubatko O Nazarov M Stepanov V . Resilience and vulnerability of Ukrainians: The role of family during the war. Probl Perspect Manage. (2024) 22:432–45. doi: 10.21511/ppm.22(1).2024.35

[15] Bonanno GA . Loss, trauma, and human resilience: have we underestimated the human capacity to thrive after extremely aversive events? Am Psychol. (2004) 59:20–8. doi: 10.1037/0003-066X.59.1.20, PMID: 14736317

[16] Lazarus RS Folkman S . Transactional theory and research on emotions and coping. Eur J Pers. (1987) 1:141–69. doi: 10.1002/per.2410010304

[17] Wesemann U Renner KH Rowlands K Köhler K Hüttermann N Himmerich H . Incidence of mental disorders in soldiers deployed to Afghanistan who have or have not experienced a life-threatening military incident—a quasi-experimental cohort study. Front Public Heal. (2024) 12:1357836. doi: 10.3389/fpubh.2024.1357836, PMID: 38584933 PMC10995976

[18] Ersen Ö Bilgiç R . The effect of proactive and preventive coping styles on personal and organizational outcomes: Be proactive if you want good outcomes. Cogent Psychol. (2018) 5:1492865. doi: 10.1080/23311908.2018.1492865

[19] Schwarzer R Knoll N . Positive coping: Mastering demands and searching for meaning. In: Positive psychological assessment: A handbook of models and measures. American Psychological Association, Washington (2003). p. 393–409. doi: 10.1037/10612-025

[20] Schwarzer R Taubert S . Tenacious goal pursuits and striving toward personal growth: proactive coping. In: Beyond coping. London: Oxford University Press (2002). p. 19–36. doi: 10.1093/med:psych/9780198508144.003.0002

[21] Greenglass E . Proactive coping. In: Frydenberg E , editor. Beyond coping: Meeting goals, vision, and challenges. Oxford University Press, London (2002). p. 37–62.

[22] Aspinwall LG Taylor SE . A stitch in time: Self-regulation and proactive coping. Psychol Bull. (1997) 121:417–36. doi: 10.1037/0033-2909.121.3.417, PMID: 9136643

[23] Aspinwall LG . Future-oriented thinking, proactive coping, and the management of potential threats to health and well-being. In: Folkman S , editor. Handbook of stress, health, and coping. Oxford University Press, New York, NY, US (2011). p. 334–65.

[24] Greenglass ER Schwarzer R Taubert S . Proactive coping inventory. PsycTESTS Dataset (1999). Washington: American Psychological Assotiation doi: 10.1037/t07292-000

[25] Southwick SM Bonanno GA Masten AS Panter-Brick C Yehuda R . Resilience definitions, theory, and challenges: interdisciplinary perspectives. Eur J Psychotraumatol. (2014) 5. doi: 10.3402/ejpt.v5.25338, PMID: 25317257 PMC4185134

[26] Calhoun LG Tedeschi RG . The foundations of posttraumatic growth: an expanded framework. In: Calhoun LG Tedeschi RG , editors. Handbook of posttraumatic growth: Research & practice. New Jersey: Lawrence Erlbaum Associates Publishers (2006). p. 3–23.

[27] Calhoun LG Tedeschi RG . Posttraumatic growth in clinical practice. London: Routledge (2012) p. 1–22.

[28] Tedeschi RG Calhoun LG . Posttraumatic growth: conceptual foundations and empirical evidence. Psychol Inq. (2004) 15:1–18. doi: 10.1207/s15327965pli1501_01

[29] Joseph S Linley PA . Trauma, recovery, and growth. New Jersey: Wiley (2008). doi: 10.1002/9781118269718

[30] Tedeschi RG Calhoun LG Cann A . Evaluating resource gain: understanding and misunderstanding posttraumatic growth. Appl Psychol. (2007) 56:396–406. doi: 10.1111/j.1464-0597.2007.00299.x

[31] Laufer A Solomon Z . Posttraumatic symptoms and posttraumatic growth among Israeli youth exposed to terror incidents. J Soc Clin Psychol. (2006) 25:429–47. doi: 10.1521/jscp.2006.25.4.429

[32] Maercker A Zoellner T . The janus face of self-perceived growth: toward a two component model of posttraumatic growth. Psychol Inq. (2004) 15:41–8.

[33] Zoellner T Maercker A . Posttraumatic growth and psychotherapy. In: Calhoun LG Tedeschi RG , editors. Handbook of posttraumatic growth: Research & practice. New Jersey: Lawrence Erlbaum Associates Publishers (2006). p. 334–54.

[34] Tedeschi RG Calhoun LG . The Posttraumatic Growth Inventory: Measuring the positive legacy of trauma. J Trauma Stress. (1996) 9:455–71. doi: 10.1007/BF02103658, PMID: 8827649

[35] Tedeschi RG McNally RJ . Can we facilitate posttraumatic growth in combat veterans? Am Psychol. (2011) 66:19–24. doi: 10.1037/a0021896, PMID: 21219044

[36] Mark KM Stevelink SAM Choi J Fear NT . Post-traumatic growth in the military: a systematic review. Occup Environ Med. (2018) 75:904–15. doi: 10.1136/oemed-2018-105166, PMID: 30377257

[37] Tsai J Sippel LM Mota N Southwick SM Pietrzak RH . Longitudinal course of posttraumatic growth among u.s. military veterans: results from the national health and resilience in veterans’ study. Depress Anxiety. (2016) 33:9–18. doi: 10.1002/da.22371, PMID: 25914061

[38] Prykhodko I Matsehora Y Kolesnichenko O Baida M Vasylkovskyi O . The psychological recovery program of Ukrainian military personnel after completing combat missions in the Russian-Ukrainian war. Cesk Psychol. (2023) 67:455–73. doi: 10.51561/cspsych.67.6.455

[39] Prykhodko I Matsehora Y Baida M . Proactive Сoping Questionnaire”: modification, approbation, psychometric indicators. Sci J Natl Acad Natl Guard “Honor Law”. (2024) 3:120–38. doi: 10.33405/2078-7480/2024/3/90/318490

[40] Zubovskiy D . Adaptation and appropriation of the Ukrainian version of the “Posttraumatic Growth Inventory” methodic. Psychol J. (2018) 7:121–35. doi: 10.31108/1.2018.7.17.8

[41] Rzeszutek M Zawadzka A Pięta M Houn A Pankowski D Kręcisz B . Profiles of resources and posttraumatic growth among cancer and psoriatic patients compared to non-clinical sample. Int J Clin Heal Psychol. (2020) 20:222–31. doi: 10.1016/j.ijchp.2020.07.004, PMID: 32994795 PMC7501453

[42] Redekop M Clark M . From life’s difficulties to posttraumatic growth: how do we get there? Psychology. (2016) 7:1451–66. doi: 10.4236/psych.2016.712144

[43] Morgan JK Desmarais SL . Associations between time since event and posttraumatic growth among military veterans. Mil Psychol. (2017) 29:456–63. doi: 10.1037/mil0000170

[44] Cederbaum JA Wilcox SL Sullivan K Lucas C Schuyler A . The influence of social support on dyadic functioning and mental health among military personnel during postdeployment reintegration. Public Health Rep. (2017) 132:85–92. doi: 10.1177/0033354916679984, PMID: 28005474 PMC5298500

[45] de Jong JTVM . Family interventions and armed conflict. In: Cross-cultural family research and practice. Elsevier Academic Press, London. (2020). p. 437–75. doi: 10.1016/B978-0-12-815493-9.00015-6

[46] Prykhodko I . The model of psychological safety of a soldier’s personality. Curr Issues Pers Psychol. (2021) 10:112–22. doi: 10.5114/cipp.2021.108684, PMID: 38013921 PMC10653560

[47] Ching C Sayeeda RJ Tarnas MC Gross N Abu-Khalil A Shaikh B . Mapping utility and applicability of research and ethics frameworks for displaced populations. Global Public Health. (2025) 20. doi: 10.1080/17441692.2025.2557322, PMID: 40975798

[48] Geller PA Hobfoll SE Dunahoo C . Women’s coping: communal versus individualistic orientation. In: International handbook of work and health psychology. New Jersey: Wiley (2009). p. 353–82. doi: 10.1002/9780470682357.ch16

