# Supplementary material for: The effect of proactive coping on posttraumatic growth among mobilized military personnel with various marital statuses after participating in combat operations
Source: Front Psychiatry. 2026 Feb 13;17:1770239. doi: 10.3389/fpsyt.2026.1770239 (PMC12946079; doi:10.3389/fpsyt.2026.1770239)
Supplement: Supplementary file 1 [file Table1.docx]

APPENDIX А

Table A1 Significance indices of the models examining the relationship between PTG (dependent variable) and proactive coping (independent variable) were marital status acting as a moderator (0 – unmarried, 1 – married).

| Model with... | F | p | R^2^ | R^2^_adjusted_ |
| --- | --- | --- | --- | --- |
| Proactive Coping | 2.635 | 0.051 | 0.033 | 0.021 |
| Reflective Coping | 3.904 | 0.010 | 0.049 | 0.036 |
| Strategic Planning | 2.044 | 0.109 | 0.026 | 0.013 |
| Preventive Coping | 3.134 | 0.026 | 0.039 | 0.027 |
| Instrumental Support Seeking | 7.039 | 0.001 | 0.084 | 0.072 |
| Emotional Support Seeking | 8.847 | 0.001 | 0.104 | 0.092 |
| Avoidance Coping | 2.968 | 0.033 | 0.037 | 0.025 |
| Proactive Coping Overall Score | 5.887 | 0.001 | 0.072 | 0.059 |
