# Supplementary material for: The effect of proactive coping on posttraumatic growth among mobilized military personnel with various marital statuses after participating in combat operations
Source: Front Psychiatry. 2026 Feb 13;17:1770239. doi: 10.3389/fpsyt.2026.1770239 (PMC12946079; doi:10.3389/fpsyt.2026.1770239)
Supplement: Supplementary file 2 [file Table2.docx]

APPENDIX B

Table B1 Beta coefficients and their significance in the models of the relationship between PTG (dependent variable) and various proactive coping, taking into account marital status (0 – unmarried, 1 – married).

| Model | | Unstandardized coefficients | | Standardized coefficients | t | p |
| --- | --- | --- | --- | --- | --- | --- |
|  |  | Beta (b) | Standard error | Beta (β) |  |  |
| 1 | (Constant) | 38.480 | 7.465 |  | 5.155 | 0.000 |
|  | Proactive Coping (PC) | 1.990 | 0.796 | 0.257 | 2.501 | 0.013 |
|  | Marital statuses (MS) (0/1) | 10.613 | 9.635 | 0.210 | 1.102 | 0.272 |
|  | Interaction term (PC*MS) | -1.647 | 1.027 | -0.333 | -1.604 | 0.110 |
| 2 | (Constant) | 32.202 | 8.216 |  | 3.920 | 0.000 |
|  | Reflective Coping (RC) | 2.537 | 0.832 | 0.333 | 3.049 | 0.003 |
|  | Marital statuses (0/1) | 14.236 | 10.153 | 0.282 | 1.402 | 0.162 |
|  | Interaction term (RC*MS) | -1.920 | 1.031 | -0.406 | -1.863 | 0.064 |
| 3 | (Constant) | 40.363 | 7.614 |  | 5.301 | 0.000 |
|  | Strategic Planning (SP) | 1.774 | 0.812 | 0.260 | 2.184 | 0.030 |
|  | Marital statuses (0/1) | 12.034 | 9.188 | 0.238 | 1.310 | 0.192 |
|  | Interaction term (SP*MS) | -1.803 | 0.971 | -0.387 | -1.856 | 0.065 |
| 4 | (Constant) | 35.860 | 8.157 |  | 4.396 | 0.000 |
|  | Preventive Coping (PreC) | 2.136 | 0.821 | 0.279 | 2.602 | 0.010 |
|  | Marital statuses (0/1) | 9.464 | 10.369 | 0.187 | 0.913 | 0.362 |
|  | Interaction term (PreC*MS) | -1.437 | 1.031 | -0.315 | -1.393 | 0.165 |
| 5 | (Constant) | 32.425 | 7.430 |  | 4.364 | 0.000 |
|  | Instrumental Support Seeking (ISS) | 2.614 | 0.775 | 0.338 | 3.372 | 0.001 |
|  | Marital statuses (0/1) | 4.112 | 9.397 | 0.081 | 0.438 | 0.662 |
|  | Interaction term (ISS*MS) | -0.788 | 1.001 | -0.155 | -0.787 | 0.432 |
| 6 | (Constant) | 35.456 | 6.572 |  | 5.395 | 0.000 |
|  | Emotional Support Seeking (ESS) | 2.157 | 0.639 | 0.313 | 3.378 | 0.001 |
|  | Marital statuses (0/1) | -3.088 | 8.689 | -0.061 | -0.355 | 0.723 |
|  | Interaction term (ESS*MS) | 0.002 | 0.866 | 0.001 | 0.002 | 0.999 |
| 7 | (Constant) | 47.068 | 8.000 |  | 5.883 | 0.000 |
|  | Avoidance Coping (AC) | 1.064 | 0.904 | 0.132 | 1.178 | 0.240 |
|  | Marital statuses (0/1) | -7.793 | 9.741 | -0.154 | -0.800 | 0.425 |
|  | Interaction term (AC*MS) | 0.517 | 1.106 | 0.098 | 0.467 | 0.641 |
| 8 | (Constant) | 24.965 | 8.642 |  | 2.889 | 0.004 |
|  | Proactive Coping Overall Score (PCOS) | 0.666 | 0.177 | 0.396 | 3.756 | 0.001 |
|  | Marital statuses (0/1) | 18.111 | 10.824 | 0.359 | 1.673 | 0.096 |
|  | Interaction term (OPCS*MS) | -0.471 | 0.222 | -0.486 | -2.116 | 0.035 |
